# Foxc1 and Foxc2 in the Neural Crest Are Required for Ocular Anterior Segment Development

**DOI:** 10.1167/iovs.16-21217

**Published:** 2017-03

**Authors:** Seungwoon Seo, Lisheng Chen, Wenzhong Liu, Demin Zhao, Kathryn M. Schultz, Amy Sasman, Ting Liu, Hao F. Zhang, Philip J. Gage, Tsutomu Kume

**Affiliations:** 1 Feinberg Cardiovascular Research Institute, Department of Medicine, Feinberg School of Medicine, Northwestern University, Chicago, Illinois, United States; 2 Department of Life Science, Research Center for Cellular Homeostasis, Ewha Womans University, Seoul, Korea; 3 Department of Ophthalmology and Visual Science, University of Michigan, Ann Arbor, Michigan, United States; 4 Department of Biomedical Engineering, Northwestern University, Evanston, Illinois, United States

**Keywords:** corneal development, neural crest, Forkhead, Pitx2, Dkk2

## Abstract

**Purpose:**

The large Forkhead (Fox) transcription factor family has essential roles in development, and mutations cause a wide range of ocular and nonocular disease. One member, *Foxc2* is expressed in neural crest (NC)-derived periocular mesenchymal cells of the developing murine eye; however, its precise role in the development, establishment, and maintenance of the ocular surface has yet to be investigated.

**Methods:**

To specifically delete *Foxc2* from NC-derived cells, conditional knockout mice for *Foxc2* (NC-*Foxc2*^−^*^/^*^−^) were generated by crossing *Foxc2^F^* mice with *Wnt1-Cre* mice. Similarly, we also generated compound NC-specific mutations of *Foxc2* and a closely related gene, *Foxc1* (NC-*Foxc1*^−^*^/^*^−^;NC-*Foxc2*^−^*^/^*^−^) in mice.

**Results:**

Neural crest-*Foxc2*^−^*^/^*^−^ mice show abnormal thickness in the peripheral-to-central corneal stroma and limbus and displaced pupils with irregular iris. The neural crest-specific mutation in *Foxc2* also leads to ectopic neovascularization in the cornea, as well as impaired ocular epithelial cell identity and corneal conjunctivalization. Compound, NC-specific *Foxc1*; *Foxc2* homozygous mutant mice have more severe defects in structures of the ocular surface, such as the cornea and eyelids, accompanied by significant declines in the expression of another key developmental factor, Pitx2, and its downstream effector Dkk2, which antagonizes canonical Wnt signaling.

**Conclusions:**

The neural crest-*Foxc2* mutation is associated with corneal conjunctivalization, ectopic corneal neovascularization, and disrupted ocular epithelial cell identity. Furthermore, Foxc2 and Foxc1 cooperatively function in NC-derived mesenchymal cells to ensure proper morphogenesis of the ocular surface via the regulation of Wnt signaling. Together, Foxc2 is required in the NC lineage for mesenchymal-epithelial interactions in corneal and ocular surface development.

The ocular surface has a central role in vision. Composed of the cornea, limbus, and conjunctiva, disorders that affect these structures or impair the lacrimal and meibomian glands' contribution to the tear film represent major causes of visual impairment. Corneal blindness due to scarring and neovascularization is the fourth leading cause of vision loss worldwide. Thus, an adequate understanding of the biological processes that contribute to ocular-surface repair, as well as the pathogenesis (and potential treatment) of ocular surface disorders, is crucially dependent on a thorough characterization of the mechanisms that participate in the normal development, maturation, and maintenance of the ocular surface, including the cornea.

The cornea, conjunctiva, and limbus form a continuous epithelial layer. In mice, the cornea begins to develop on or around E10.5, immediately after the lens vesicle separates from the surface ectoderm. The surface ectoderm then becomes the presumptive corneal epithelium, and neural crest (NC)-derived mesenchymal cells migrate into the developing eye, where they contribute to formation of the anterior segment, including the corneal stroma and endothelium.^1,2^ The corneal epithelium initially consists of just one to two cell layers but proliferates and differentiates after birth into stratified squamous epithelium, which is composed of six to eight cell layers. In adult mice, the corneal epithelium is maintained by slow-dividing basal cells, which replace the superficial epithelial cells and are themselves replenished by stem cells in the corneal/limbal epithelium^3,4^ (see below). Importantly, the development, maturation, and maintenance of the ocular surface are dependent on interactions between the mesenchyme and epithelium that are mediated by signaling mechanisms such as the canonical Wnt pathway. However, the cellular and molecular components responsible for coordinating these signal transduction networks are largely unknown, and whether NC-derived mesenchymal cells contribute to these processes in the ocular surface has yet to be fully elucidated.

Corneal transparency is critical for proper ocular surface function and can be impaired by corneal neovascularization (i.e., the pathologic growth of corneal vessels). Corneal neovascularization can occur in response to a wide range of insults, including infections, trauma, chemical burns, and inflammation, as well as during recovery from corneal transplantation surgery, where the growth of new blood vessels often determines corneal graft survival.^5^ Corneal transparency is known to be regulated by soluble VEGF receptor sVEGFR-1 (sFlt-1),^6^ and the abnormal growth of corneal blood vessels is accompanied by VEGF-dependent corneal conjunctivalization.^7,8^ Conjunctivalization of the corneal surface, accompanied by vascularization, the appearance of goblet cells, and an irregular and unstable epithelium, is associated with limbal stem cell deficiency (LSCD), a severe ocular disease that is characterized by abnormal corneal epithelial maintenance.^9,10^ Limbal stem cell deficiency can develop from a variety of pathologic or congenital causes and often results in the loss of vision.

Recent studies in mutant mice have shown that the homeodomain transcription factor *Pitx2* and its downstream effector *Dkk2* play a pivotal role in the specification and maintenance of cell fate in corneal and conjunctival ectoderm.^11,12^ Axenfeld-Rieger Syndrome (ARS), an autosomal dominant disorder, encompasses a range of congenital malformations affecting the anterior segment of the eye including corneal opacity^13–15^ and has been linked to *PITX2* and the Fox transcription factor *FOXC1*. We have previously shown that NC-specific *Foxc1* heterozygous and homozygous mutant (NC-*Foxc1*^+/−^ and NC-*Foxc1*^−^*^/^*^−^) mice recapitulate the corneal phenotypes observed in patients with ARS secondary to heterozygous *FOXC1* mutations.^16^ Inactivating mutations in a closely related Fox family gene, *FOXC2*, are responsible for the autosomal dominant syndrome Lymphedema-distichiasis, which is characterized by the obstruction of lymphatic drainage in the limbs and by the growth of extra eyelashes (distichiasis), which arise from the meibomian glands (OMIM 153400).^17,18^ Notably, the affected individuals also have ocular problems such as corneal irritation (including, in some cases, corneal ulceration), recurrent conjunctivitis, and photophobia. Murine *Foxc2* is expressed in the ocular mesenchyme of NC origin.^19^ However, the precise roles of Foxc2 in ocular development, including the formation of the cornea, remain incompletely understood,^20^ mainly due to the midgestation lethality of global *Foxc2* knockout mice.

In this study, we show that an NC-specific mutation of *Foxc2* in mice leads to abnormal formation of the cornea, corneal neovascularization and conjunctivalization, and impaired ocular epithelial cell identity. We further show that compound, NC-specific mutant mice for *Foxc2* and a closely related gene, *Foxc1*,^16,21^ have more severe eye defects, including the absence of the cornea. More importantly, lack of both *Foxc* genes in the NC-lineage results in significant declines in the expression of another key developmental factor, Pitx2, and its downstream effector Dkk2, which antagonizes canonical Wnt signaling. Thus, these results indicate that the expression of Foxc2 in NC-derived cells is crucial for corneal development, differentiation, and avascularity, as well as for maintaining corneal epithelial identity.

## Methods

### Animals

Conditional floxed *Foxc2*^F^ and *Foxc1*^F^;*Foxc2*^F^ mice were used.^22^ *Wnt1-Cre*;*Foxc2*^F/F^ (NC-*Foxc2*^−/−^) and *Wnt1-Cre*;*Foxc1*^F/F^;*Foxc2*^F/F^ (NC-*Foxc1*^−/−-^;NC-*Foxc2*^−/−^) mice were generated by crossing *Foxc2*^F^ and *Foxc1*^F^;*Foxc2*^F^ mice with *Wnt1-Cre* mice^23^ (gift from Andrew McMahon, Harvard University), respectively. For cell fate mapping, *Wnt1-Cre;Foxc2*^F^ mice were crossed with R26R reporter mice.^24^ Embryonic age was determined by defining noon of the day of the vaginal plug as embryonic day 0.5 (E0.5). All experimental procedures complied with the ARVO Statement for the Use of Animals in Ophthalmic and Vision Research, and the experimental protocols used in this study were approved by the Institutional Animal Care and Use Committee at Northwestern University.

### Tissue Processing

Whole embryos and eyeballs were fixed in 4% paraformaldehyde (PFA) for 2 hours at 4°C, dehydrated with methanol, embedded in paraffin, and cut into 8-μm sections. For frozen sections, whole embryos and eyeballs were fixed in 4% PFA, immersed in 30% sucrose (wt/vol in PBS), and then embedded in OCT Compound (Tissue-Tek) and frozen in an ethanol/dry ice bath. Frozen tissues were cut into 8-μm sections.

### Histologic and Immunohistochemical Analyses

Paraffin sections were stained with hematoxylin and eosin (H&E) and subject to immunohistochemistry. Periodic acid Schiff (PAS; 395B, Sigma-Aldrich Corp., St. Louis, MO, USA) stain was performed on paraffin sections of eyes according to the manufacturer's protocol.

For fate mapping of NC cells, whole mount X-gal staining of E11.5 and E13.5 embryos was performed as previously described.^16^

For immunohistochemical analyses, paraffin sections were submerged in citrate buffer, pH 6, and heat treated in a rice cooker for 20 minutes, then blocked with 10% normal donkey serum (Sigma-Aldrich Corp.) in PBS or 10% normal goat serum (Sigma-Aldrich Corp.) in PBS with 0.1% Triton X-100 (Sigma-Aldrich Corp.). Sections were incubated in blocking buffer with antibodies overnight at 4°C, washed with PBS/0.1% Tween 20, and then incubated with secondary antibodies conjugated to either AlexaFluor488 or AlexaFluor568 (Invitrogen, Carlsbad, CA, USA) for 1 hour at room temperature, counterstained with 4′,6-diamidino-2-phenylindole (DAPI), and mounted with Fluoromount aqueous mounting medium (Sigma-Aldrich Corp.). Primary antibodies used include Keratin 12 (L-15, Santa Cruz Biotechnology, Santa Cruz, CA, USA), Keratin 14 (clone MK14, Covance, Princeton, NJ, USA), Keratin 15 (Covance), and p63α (#4892, Cell Signaling Technology, Danvers, MA, USA).

For immunostaining for PITX2 and AP2-β (TFAP2B), paraffin sections were processed and stained with antibodies against PITX2 (gift from Tord Hjalt, Lund University, Sweden) or AP-2β (Abnova, Walnut, CA, USA), as described previously.^25^

### In Situ Hybridization

Paraffin sections were processed for in situ hybridization to detect *Dkk2* expression in the developing eye using an antisense riboprobe to *Dkk2*, as previously described.^12,26^

### Corneal Flat-Mount Immunostaining

Corneal flat-mount immunostaining was performed as previously described.^16^ Briefly, enucleated eyes were fixed in 4% PFA for 20 minutes at 4°C to avoid overfixation of the cornea, rinsed in PBS, and cut in half. Following removal of the extra tissues including lens and iris, the cornea was cut radially to facilitate flat mounting.

For flat-mount immunostaining to detect blood and lymphatic vessels, the corneas were placed in −20°C 100% methanol for 30 minutes and permeabilized with PBS/1% Triton X-100 for 20 minutes at 4°C, and blocked with 5% donkey serum (Sigma-Aldrich Corp.) for 20 minutes, and then stained with anti-Lyve-1 antibodies (Abcam, Cambridge, MA, USA) overnight at 4°C. Corneas were then washed with PBS/0.1% Tween 20 and then stained with PE-conjugated anti-CD31 (BD Biosciences Phamingen, San Diego, CA, USA; clone MEC 13.3) and AlexaFluor488 donkey anti-rabbit IgG (Invitrogen). Corneas were flat mounted and images were recorded on a Zeiss Axio Observer D1.

### Anterior Spectral-Domain (SD) Optical Coherence Tomography (OCT) Angiography

Optical coherence tomography is a depth-resolved imaging technique with great applications in both clinical settings and laboratory investigations. By combining low-coherence light illumination and interferometric signal detection and analysis, OCT achieves micrometer level axial resolution with high sensitivity. In our work, we employed a broadband fiber output light source (center wavelength: 840 nm; bandwidth: 95 nm; IPSDW0825C-0314, Inphenix, Livermore, CA, USA), and a 50×50 fiber coupler (OZ Optics, Carp, ON, Canada) for interferometry. Interference signals between the sample and reference arm were sampled with a homemade spectrometer, comprising a grating (Wasatch, 1200 lines/mm), an imaging lens (Thorlabs, Newton, NJ, USA; focal length =150 mm) and a line-camera (Basler, Ahrensburg, Germany; 2048 pixels, 140 KHz highest sampling rate, 50 KHz in our current experiment). The lateral resolution was 5.6 μm, the axial resolution was 3.2 μm, and the imaging depth was approximately 1200 μm.

Optical coherence tomography angiography (OCTA) can map vascular network using overlapping B-scan imaging at single B-scan location. In this study, we applied OCTA to evaluate corneal neovascularization in vivo; the imaging region of interest was decided to be central cornea to avoid confusion from limbal vasculature at corneal boundary. We repeated five B-scan images at every location, with 400 A-lines in each B-scan and 512 discrete B-scan positions in total to produce a volumetric corneal map (2.5 mm by 2.5 mm). To visualize vasculature, we first analyzed OCTA data^27^ by evenly dividing the 95-nm bandwidth into four nonoverlapping subbands and reconstructing corresponding B-scan images from each subband. Then, within each subband, we took the complex signal differences between sequential B-scans at the same location to generate vasculature imaging.^28–30^ Results from four subbands were averaged, and we repeated this process across 512 discrete B-scan locations to produce the final angiography map.

Before imaging, mice were anesthetized by a cocktail of Ketamine (87 mg/kg body weight) and Xylazine (13 mg/kg body weight)^31^ through intraperitoneal injection and restrained in a homemade animal holder with six degrees of adjustment freedom. Artificial tear (Systane, Alcon Laboratories, Inc., Fort Worth, TX, USA) were applied every other minute to prevent corneal dehydration.

## Results

### NC-Specific Deletion of *Foxc2* in Mice Results in Multiple Ocular Defects

Global *Foxc2*^−/−^ mice die prenatally (at ∼E16.5) with severe cardiovascular abnormalities.^32–34^ By breeding our conditional *Foxc2* (*Foxc2*^F^) mice^22^ with *Wnt1-Cre* mice that express Cre recombinase under the control of *Wnt1* regulatory elements^23^ and are widely used in the study of the development of the NC, we generated NC-specific *Wnt1-Cre;Foxc2^F/F^* (NC-*Foxc2*^−/−^) mice. Importantly, NC-*Foxc2*^−/−^ mice survived until adulthood and were fertile; thus, these mice provide a unique (and unanticipated) model for studying the congenital ocular defects associated with NC-lineage mutations.

**Figure 1:**
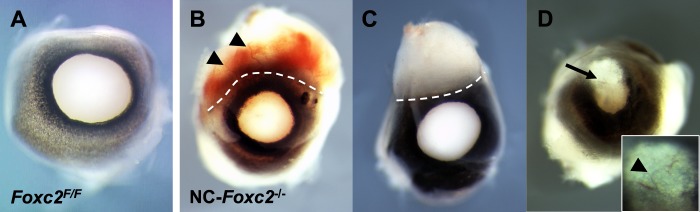
Representative ocular phenotypes in NC-specific *Foxc2*^−/−^ mutant mice at P0. (**A**–**D**) The eyeballs of control (*Foxc2^F/F^*) (**A**) and NC-*Foxc2*^−/−^ (**B**–**D**) mice at P0. Neural crest-*Foxc2*^−/−^ mice exhibit smaller eyes with irregular iris and abnormal morphology in the cornea and conjunctiva. The peripheral cornea–limbus–conjunctiva regions had abnormal tissue attachment (**B**, **C**, *dotted lines*) with superficial hemorrhagic blood vessels (**B**, *arrowheads*). The eyes of NC-*Foxc2*^−/−^ mutants also had a corneal plaque (*arrow*) with blood vessels (**D**, *inset*, *arrowhead*).

We first carried out fate mapping of NC-derived cells in NC-*Foxc*2^−/−^ mice. The neural crest cells of the NC-*Foxc2*^−/−^ mice at E11.5 to E13.5 appeared to migrate normally and participate in early eye development (Supplementary Fig. S1), but their corneas failed to develop normally (Fig. 1). Neural crest-*Foxc2*^−/−^ mice at postnatal day 0 (P0) had smaller eyes with irregular iris and abnormal morphology in the cornea and conjunctiva including an uncharacteristic tissue attachment in the peripheral cornea–limbus–conjunctiva regions (Figs. 1B, 1C) and an opaque corneal plaque formation with blood vessels (Fig. 1D), whereas they were born with closed eyelids (data not shown). Interestingly, NC-*Foxc2*^−/−^ mice exhibited the conjunctival hyperemia with superficial hemorrhagic blood vessels (Fig. 1B). We similarly observed the outgrowth of blood vessels in the cornea of global *Foxc2*^−/−^ embryos at E15.5 (Supplementary Fig. S2).

**Figure 2:**
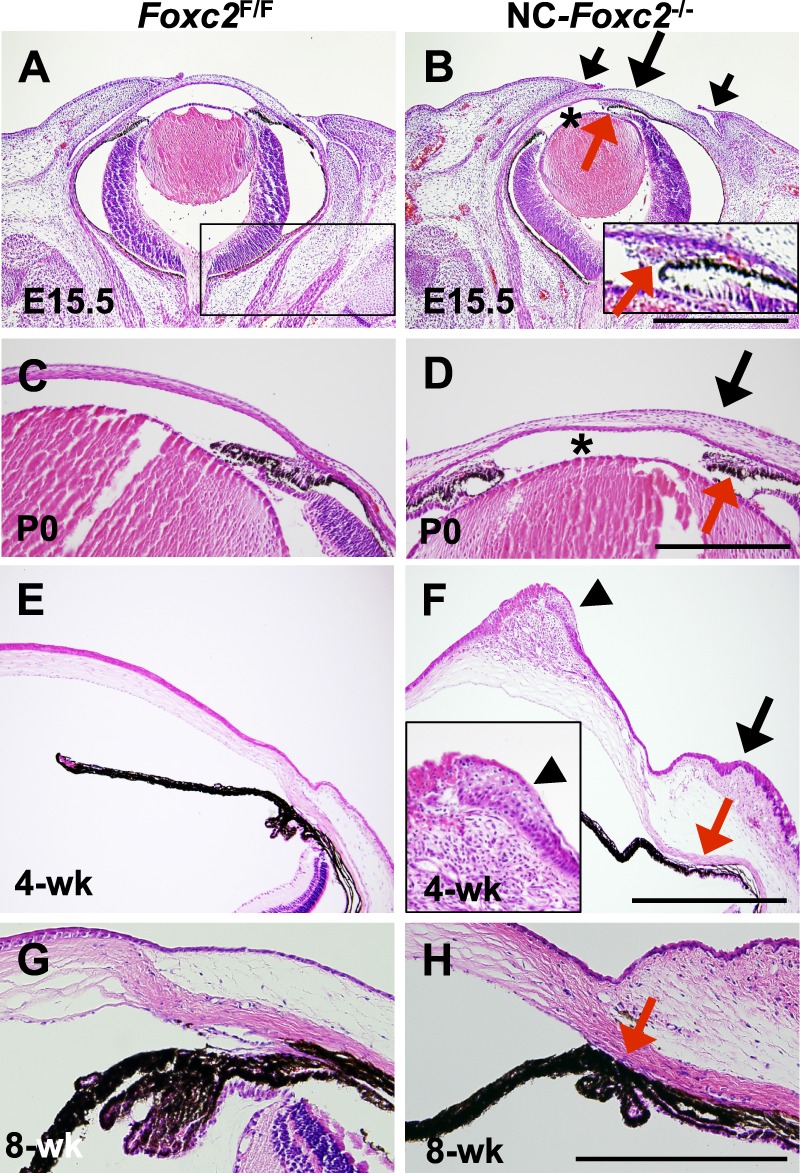
Hyperplasia of cornea and conjunctiva in NC-specific *Foxc2*^−/−^ mutant embryos and mice. (**A**–**H**) H/E stain of eyes of control (*Foxc2^F/F^*) and NC-*Foxc2*^−/−^ mice at E15.5, P0, 4 weeks, and 8 weeks. In contrast to control mice (**A**, **C**, **E**), NC-*Foxc2*^−/−^ mice had abnormally thickened peripheral cornea and conjunctiva (*arrows*) at E15.5 (**B**), P0 (**D**), and 4 weeks (**F**), but their eyelids were normally developed (**B**, *short arrows*). While the anterior chamber (*asterisk*) of NC-*Foxc2*^−/−^ mice at E15.5 and P0 was present, the pupil was relatively displaced to one side (**B**). Neural crest-*Foxc2*^−/−^ mice had hypoplastic trabecular meshwork (**B**, *inset*; **D**), defective iridocorneal angle (**D**), hypoplastic and underdeveloped ciliary process (**F**, **G**) as indicated by *red arrows*, compared with those of control mice (**A**, **C**, **E**, **G**). Note hyperplasia of corneal stroma and epithelium with aberrant accumulation of mesenchyme-like cells (**F**, *inset*, *arrowhead*) in NC-*Foxc2*^−/−^ mice at 4 weeks. *Scale bars*: 100 μm.

Neural crest-*Foxc2*^−/−^ mice developed an anterior chamber (Figs. 2B, 2D). However, the peripheral corneas and conjunctiva of NC-*Foxc2*^−/−^ mice were abnormally thick at E15.5 and P0 and became much thickened at 4 weeks, showing hyperplasia of the conjunctiva (Figs. 2B, 2D, 2F). Neural crest-*Foxc2*^−/−^ mice had hyperplastic corneas with epithelial breakdown and aberrant accumulation of mesenchymal-like cells (Fig. 2F), suggesting congenital defect caused by *Foxc2* deficiency as shown in Figure 1D, not just due to eye dryness. The neural crest-*Foxc2*^−/−^ mutation was also associated with hypoplasia of the presumptive trabecular meshwork, defects in the iridocorneal angle, and underdeveloped ciliary processes (Figs. 2B, 2D, 2F, 2H).

### NC-Specific *Foxc2*^−/−^ Mice Exhibit Corneal Neovascularization

**Figure 3:**
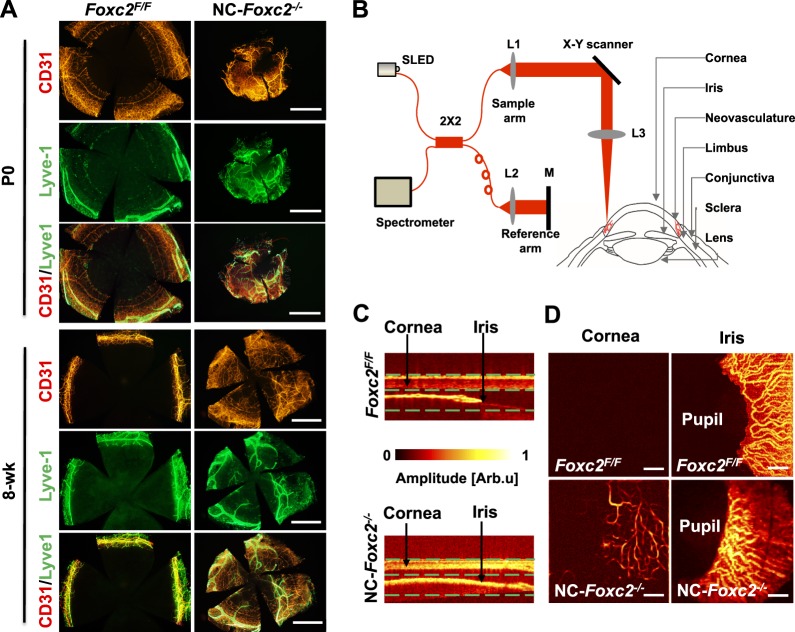
Neural crest-specific *Foxc2*^−/−^ mutant mice exhibit corneal neovascularization and lymphangiogenesis. (**A**) Corneal flat mounts of P0 and 8-week-old mice for the blood vessel marker CD31 (*red*) and lymphatic vessel marker Lyve-1 (*green*). *Foxc2*^F/F^ corneas were avascular, whereas NC-*Foxc2*^−/−^ mutants had abnormal blood and lymphatic vessels in the cornea at P0 and 8 weeks. (**B**–**D**) Anterior SD-OCT system for monitoring corneal neovascularization in adult *NC-Foxc2*^−/−^ mice. (**B**) Schematic illustration of the SD-OCT experimental system and the geometry of the corneal imaging. L1 to L3 are lens (L3 focal length, 40 mm); M, mirror. (**C**) Optical coherence tomography B-scan anatomical images of cornea and iris from *Foxc2^F/F^* and NC-*Foxc2*^−/−^ mice at 7 weeks. (**D**) Reconstruction of OCT B-scan images of cornea and iris from *Foxc2^F/F^* and NC-*Foxc2*^−/−^ mice at 7 weeks. Signals from cornea and iris, indicated by (**C**), were reconstructed by the OCTA for visualization of blood vessels. Note an abnormal vascular network in the cornea sprouting from the limbus and disrupted iris vessels in NC-*Foxc2*^−/−^ mice, compared with avascular cornea and clear iris vascular network in *Foxc2^F/F^* mice. *Scale bars*: 200 μm.

Since we found the ectopic outgrowth of blood vessels in the cornea of both the global *Foxc2*^−/−^ (Supplementary Fig. S2) and NC-*Foxc2*^−/−^ mice (Fig. 1D), we further investigated the process of abnormal neovascularization in the cornea of NC-*Foxc2*^−/−^ mice by performing corneal flat mount immunostaining for CD31 and Lyve-1. Ectopic outgrowth of CD31^+^ blood vessels as well as Lyve-1+ lymphatic vessels from the limbus was observed in P0 and 8-week-old NC-*Foxc2*^−/−^ mice (Fig. 3A). These data suggest that the expression of Foxc2 in NC-lineage cells maintains corneal transparency by preventing ectopic blood and lymphatic vessel growth.

We recently assessed corneal neovascularization by adapting optical-resolution photoacoustic microscopy (OR-PAM) for use in adult mice^35^ and then developed an even more advanced anterior SD-OCT system (Fig. 3B). Spectral-domain optical coherence tomography angiography is a noncontact, noninvasive, blood-flow based technology. Our home-built anterior SD-OCT system uses a light source with a center wavelength of 840 nm and a bandwidth of 95 nm, which produces an axial resolution of 3 μm, and has an A-line rate of 70 kHz. We used this system^36,37^ to obtain images of corneal vessel outgrowth in control (*Foxc2^F/F^*) and NC-*Foxc2*^−/−^ mice at 7 weeks. A network of new corneal vessels was clearly visible in the NC-*Foxc2*^−/−^ eyes, and the disrupted vascular network of the iris, which is located directly below the corneal vessels, was acquired simultaneously (Fig. 3D). The newly formed corneal vessels were aligned radially toward the center of the cornea, suggesting that neovascularization begins at the periphery and propagates inward. Vessels were not observed in the corneas of eyes from the control mice (Fig. 3D).

### Ocular Surface Epithelial-Cell Maintenance Is Disrupted in NC-*Foxc2*^−/−^ Mice

**Figure 4:**
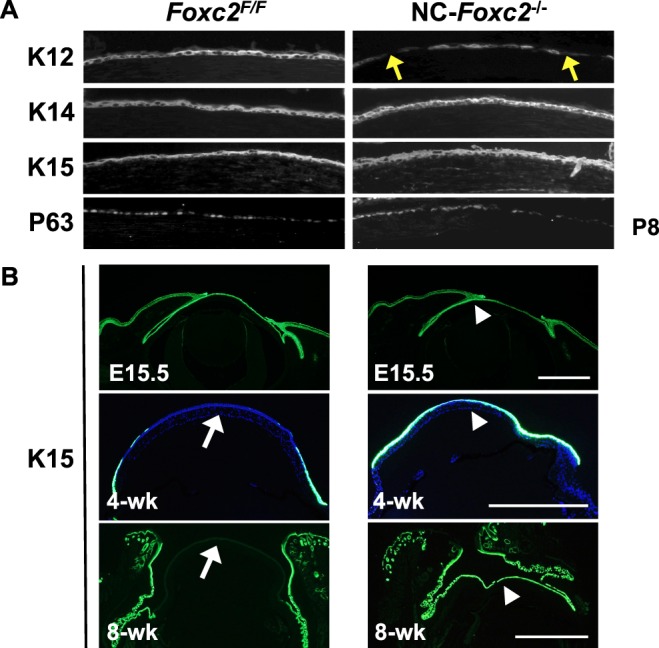
Altered cell fate in the corneal epithelium of NC-*Foxc2*^−/−^ mice. (**A**, **B**) *Foxc2* deletion altered differentiation of the corneal epithelium at P8, 4 weeks, and 8 weeks. (**A**) Corneas from P8 *Foxc2^F/F^* and NC-*Foxc2*^−/−^ mice were stained with antibodies for Keratins (K) 12, 14, and 15, and P63, a progenitor cell marker. K12 expression was decreased in NC-*Foxc2*^−/−^ corneal epithelium (*arrows*) at P8. (**B**) Altered expression of K15, a limbal/conjunctiva epithelial marker, in the corneal epithelium of NC-*Foxc2*^−/−^ mice at 4 weeks and 8 weeks. K15 expression in *Foxc2^F/F^* mice was not detected in the central corneas at 4 weeks and the entire corneas at 8 weeks (*arrows*), while its expression was persistent in the corneas of NC-*Foxc2*^−/−^ mice from E15.5 through 8 weeks (*arrowheads*). *Scale bars*: 200 μm.

The components of corneal and conjunctival epithelium that are derived from the corneal surface ectoderm begin to undergo specification at approximately E12.5.^38^ Thus, we examined whether *Foxc2* deficiency in the ocular stroma may interfere with ocular epithelial identity and maintenance by analyzing the expression of a panel of epithelial markers at E15.5, P8, and 8 weeks. Notably, the expression of cytokeratin 12 (K12), which is a cornea-specific and differentiation-dependent marker,^39,40^ was significantly lower in NC-*Foxc2*^−/−^ mice than in control (*Foxc2^F/F^*) mice at P8 (Fig. 4A), while the expression of cytokeratin 14 (K14), a maker for ocular surface (corneal/limbal/conjunctival) epithelium,^40^ in the two groups was similar throughout ocular development (Fig. 4A, Supplementary Fig. S3). This implies impaired formation of the corneal epithelium in NC-*Foxc2*^−/−^ mice. Furthermore, the expression of cytokeratin 15 (K15), which is present in the epithelium of the cornea–limbus–conjunctiva in prenatal embryos and normally restricted to the epithelium of the limbus and conjunctiva in adult mice, was unexpectedly observed in the central corneal epithelium of adult NC-*Foxc2*^−/−^ mice (Fig. 4B). Thus, K15 expression persisted throughout the differentiation and maturation of the corneal epithelium in NC-*Foxc2*^−/−^ mice.

The meibomian glands are sebaceous glands located at the rim of the eyelids that lack hair follicles and secrete the lipid that forms the outermost lipid layer of the tear film, which prevents the tears from evaporating. Lymphedema-distichiasis syndrome (OMIM 153400) is an autosomal dominant disease caused by mutations in *FOXC2*, and its most consistent feature, distichiasis^41^ (i.e., the presence of aberrant, extra eyelashes, which grow from the meibomian glands). The role of NC-derived mesenchymal cells in the development of the meibomian glands remains poorly understood.^4^ We found that adult NC-specific *Foxc2*^−/−^ mice displayed hypoplasia of the meibomian glands (Supplementary Fig. S4) and extra eyelashes (data not shown), suggesting the importance of Foxc2 expression in NC-derived mesenchymal cells for meibomian gland development.

**Figure 5:**
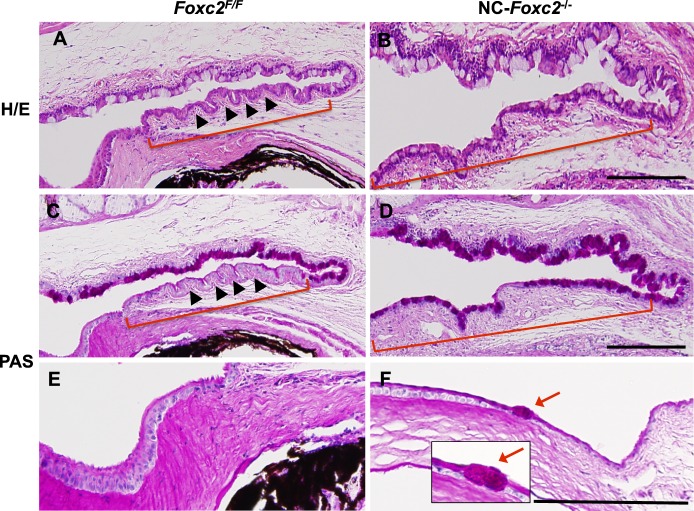
Ectopic goblet cell formation in the corneal epithelium of adult NC-*Foxc2*^−/−^ mice. (**A**–**F**) H/E (**A**, **B**) and PAS (**C**–**F**) stain of eyes in control (*Foxc2^F/F^*) and NC-*Foxc2^−/−^* mice at 8 weeks. Periodic acid Schiff stain was performed to detect mucin-secreting goblet cells. Neural crest-*Foxc2*^−/−^ mice had defective stratification of columnar epithelium (**B**, **D**, *brackets*) in conjunctiva, compared with normal stratified columnar shape without goblet cells (**A**, **C**, *brackets*, *arrowheads*) in the conjunctival epithelium of control mice. Neural crest-*Foxc2*^−/−^ mice displayed conjunctival goblet cell expansion (**B**, **D**, *brackets*) and an ectopic goblet cell (**F**, *red arrows*, *inset*) in the peripheral corneal epithelium. *Scale bars*: 100 μm.

Compared to observations in control mice (Figs. 5A, 5C), the conjunctiva of NC-*Foxc2*^−/−^ mice contained much less stratified columnar epithelium and a notably larger number of PAS+ goblet cells (Figs. 5B, 5D). Ectopic PAS+ goblet cells were also present in the peripheral corneal epithelium of NC-*Foxc2*^−/−^ mice (Fig. 5F), which is consistent with corneal conjunctivalization and the impairment of corneal epithelial cell identity, because NC-derived periocular mesenchymal cells contribute to the mesenchyme layer of the limbus^19^ and conjunctiva,^1,42^ as well as the corneal stroma. Taken together, these observations suggest that *Foxc2* deficiency in NC-derived cells leads to defects in establishment and maintenance of corneal epithelial identity.

### Abnormal Eye Development in NC-Specific Compound *Foxc1*^−/−^;*Foxc2*^−/−^ Mice

**Figure 6:**
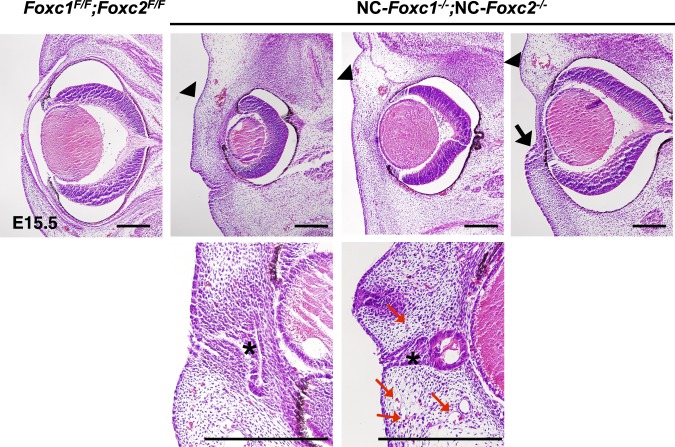
Abnormal development of the anterior eye segment in compound NC-*Foxc1*^−/−^;NC-*Foxc2*^−/−^ embryos. H/E stain of control (*Foxc1^F/F^*; *Foxc2^F/F^*) and NC-*Foxc1*^−/−^*;*NC-*Foxc2*^−/−^ eyes at E15.5. Anterior-segment development in NC-*Foxc1*^−/−^*;*NC-*Foxc2*^−/−^ embryos was severely impaired (*arrowheads*) with lack of the anterior chamber, fusion of the cornea epithelium and the ocular surface ectoderm (*asterisks*, *bottom panels*), and aberrant infiltration of capillaries with red blood cells (*red arrows*). Note that the eyelids either failed to form or were underdeveloped (*arrow*) in NC-*Foxc1*^−/−^*;*NC-*Foxc2*^−/−^ mutants. *Scale bars*: 100 μm.

Both Foxc1 and Foxc2 are expressed in NC-derived periocular mesenchymal and conjunctival epithelial cells during eye development.^12,21,43,44^ Although Foxc1 is expressed in both the NC-derived and the mesoderm-derived periocular mesenchyme of mice, the majority of Foxc1+ cells are derived from NC cells,^19^ and we have previously shown that *Foxc1* deficiencies in murine NC-lineage cells lead to anterior-segment defects (including corneal neovascularization) and similar to those in global *Foxc1* mutant embryos.^16^ Because *global* compound *Foxc1*;*Foxc2* homozygous mutant embryos die at ∼E9.5,^33^ attempts to determine how the two genes may function cooperatively during early eye development have been hampered. Remarkably, our new studies revealed that NC-specific homozygous compound *Foxc1*;*Foxc2* mutant (NC-*Foxc1*^−/−^;NC-*Foxc2*^−/−^) embryos at E15.5 developed severe defects in structures of the ocular surface, such as the cornea and eyelids, accompanied by possible fusion of corneal epithelium and ocular surface ectoderm, as well as aberrant infiltration of capillaries with red blood cells at E15.5 (Fig. 6). Compound NC-hetero/homozygous (NC-*Foxc1^+/^*^−^;NC-*Foxc2*^−/−^ and NC-*Foxc1*^−/−^;NC-*Foxc2^+/^*^−^) mice at P0 had smaller eyes, irregular iris, and narrow notched pupil shape. The ocular phenotypes of the compound NC-*Foxc1*^−/−^;NC-*Foxc2^+/^*^−^ mice appeared more severe than those of the compound NC-*Foxc1^+/^*^−^;NC-*Foxc2*^−/−^ mice (Supplementary Fig. S5). The compound NC-*Foxc1*^+/−^; NC-*Foxc2*^−/−^ mice had more severe eye defects than either NC-*Foxc1*^+/−^ mice^16^ or NC-*Foxc2*^−/−^ mice (Fig. 1). These data suggest that early development of the anterior segment appears to be critically dependent on the combined number of functional *Foxc1* and *Foxc2* genes (i.e., the *Foxc* gene dose) in NC-derived periocular mesenchymal cells. Thus, our newly generated compound NC-*Foxc1*;NC-*Foxc2* homozygous mutants provide the first opportunity to characterize how the *Foxc* genes may cooperatively regulate signaling (e.g., the canonical Wnt pathway) in NC-lineage cells during early eye development (see below).

### Reduction of Pitx2/Dkk2 Expression in Compound NC-*Foxc1/c2* Mutants

**Figure 7:**
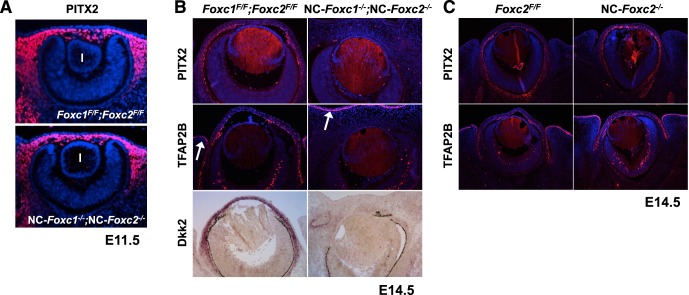
Expression of Pitx2, Tfap2b, and *Dkk2* in the eyes of NC-*Foxc* mutant embryos. (**A**) Normal Pitx2 expression in the periocular mesenchyme of compound NC-*Foxc1*^−/−^;NC-*Foxc2*^−/−^ embryo at E11.5. l, lens. (**B**) PITX2 and its target TFAP2 were present in the periocular mesenchyme, as well as corneal stroma and endothelium layers in the control at E14.5, but they were not detected in compound NC-*Foxc1*^−/−^;NC-*Foxc2*^−/−^ embryos. TFAP2B was also strongly expressed in surface ectoderm at similar levels in the control and compound NC-*Foxc1*^−/−^;NC-*Foxc2*^−/−^ eyes (*arrows*). In situ hybridization was used to detect *Dkk2*, a direct target of PITX2, and its expression within cornea stroma and periocular mesenchyme was completely lost in compound NC-*Foxc1*^−/−^;NC-*Foxc2*^−/−^ embryo. (**C**) PITX2 and TFAP2B were normally present in the eye of NC-*Foxc2*^−/−^ embryo.

Although both *FOXC1* and *PITX2* mutations in humans are associated with ARS phenotypes,^13–15^ the ocular defects observed in NC-specific *Pitx2* mutant mice are somewhat different from those seen in either single NC-*Foxc* or compound NC-*Foxc1*;NC-*Foxc2* homozygous mutants. For example, NC-*Pitx2* mutants have a shortened optic stalk with displacement of the optic cup to a midline position.^25^ We found that the compound NC-*Foxc1*^−/−^;NC-*Foxc2*^−/−^ mutation did not alter Pitx2 expression in the periocular mesenchyme at E11.5 (Fig. 7A); but by E14.5, levels of Pitx2, as well as its downstream effectors *Dkk2*^11,12^ (a canonical Wnt signaling antagonist) and the AP-2β transcription factor Tfap2b,^45^ were significantly lower in compound NC-*Foxc1*^−/−^;NC-*Foxc2*^−/−^ embryos than in control embryos (Fig. 7B). The fact that Pitx2 expression in NC-derived mesenchymal cells is present in the NC-*Foxc1*^−/−^;NC-*Foxc2*^−/−^ embryos at E11.5 (Fig. 7A) provides compelling evidence that the NC migration in these mutants is sufficient to reach the eye, including the cornea. It should be noted that Tfap2b was normally expressed in the surface ectoderm of the compound NC-*Foxc1*^−/−^;NC-*Foxc2*^−/−^ embryos (Fig. 7B), which indicates that the Wnt1-Cre driver is specific to the NC-derived periocular mesenchyme, not the surface ectoderm. By contrast, NC-*Foxc2* mutation alone did not alter expression levels of Pitx2 and Tfap2b at E15.5 compared to control embryos (Fig. 7C). Taken together, these results indicate that Foxc1 and Foxc2 are not required for the induction of Pitx2 expression in the periocular mesenchyme, but they appear to be necessary for maintaining Pitx2 expression during eye development.

## Discussion

In this study, we show that Foxc2 is required in the NC-lineage for corneal development and the establishment and maintenance of ocular epithelial identity. Heterozygous *FOXC2* mutations do not produce the malformations observed in patients with *FOXC1/PITX2*-associated ARS,^46,47^ but they result in ocular anterior segment anomalies such as iris hypoplasia, pupil displacement, and decreased anterior segment size,^46^ which are milder than those caused by *FOXC1* mutations. Of note, NC-specific *Foxc2* mutant mice closely recapitulate the individuals with global *FOXC2* mutations. Furthermore, our study using NC-specific compound *Foxc1/c2* homozygous mutant mice identifies the molecular and genetic networks involving the Pitx2/Dkk2 cascade in ocular development. These results will help elucidate the fundamental mechanisms that regulate the formation and maintenance of the ocular surface and understand how disruption of these mechanisms leads to defects in the ocular surface and cornea.

### Involvement of Foxc2 in Corneal Development and the Establishment and Maintenance of Ocular Epithelial-Cell Identity

In mice, global homozygous knockout mutations of *Foxc2* are embryonic lethal, and the precise function(s) of Foxc2 in the eye have yet to be fully elucidated. Our new studies show that when the mutation is restricted to NC cells, the animals survive to adulthood and are fertile. Our data provide cogent evidence that Foxc2 is required in NC-derived cells for development of the cornea and ocular surface, corneal avascularity, meibomian gland development, and corneal epithelial cell identity.

The surface ectoderm is specified into corneal epithelium in response to signaling from the underlying mesenchyme,^11,48^ and several lines of evidence also suggest that NC-derived cells contribute to the mesenchyme layer of the limbus^19^ and conjunctiva,^1,42^ as well as the corneal stroma. Despite Foxc2 not normally being expressed in the corneal epithelium,^12,21,43,44^ we found that the maintenance of corneal epithelial fate is disrupted in NC-specific *Foxc2*^−/−^ mice (Fig. 4), and these mutant mice also exhibit corneal conjunctivalization (Fig. 5). Abnormal corneal epithelial maintenance and corneal conjunctivalization are two primary characteristics associated with LSCD,^49^ a severe ocular disease that often results in blindness and can be caused both by the loss/dysfunction of limbal stem cells and by alterations of the limbal microenvironment. Thus, the NC*-Foxc2* deficiency may contribute to corneal conjunctivalization through (1) the loss or dysfunction of limbal epithelial stem cells, (2) aberrations in epithelial differentiation that favor limbal epithelium at the expense of corneal epithelium,^50^ or (3) both. Corneal conjunctivalization is mediated, at least in part, by VEGF signaling,^7,8^ and our previous studies show that the lack of NC-lineage Foxc1 expression enhances VEGF signaling (leading to corneal angiogenesis) in the cornea.^16^ Therefore, Foxc2 may govern corneal epithelial maintenance by regulating VEGF signaling.

### Cooperative Role of *Foxc1* and *Foxc2* in Corneal Development

Our studies demonstrate that NC-Foxc1 and NC-Foxc2 expression cooperatively regulate early development of the cornea by interacting with the Pitx2/Wnt signaling cascade (Fig. 7, Supplementary Fig. S6). Remarkably, compound NC-double homozygous *Foxc1/c2* mutant embryos at E14.5 exhibit undetectable levels of Pitx2 and its downstream effectors, *Dkk2* and AP-2β, which is reinforced by our finding that the double mutants closely phenocopy both the global and NC-specific mutant mice for *Pitx2*.^25,51^ During early eye development, *Pitx2* and *Foxc1* expression in the periocular mesenchyme is dependent on retinoic acid signaling.^52,53^ Our study indicates that Foxc1 and Foxc2 are not required for the induction of Pitx2 expression in early eye development, but they appear to be necessary for maintaining Pitx2 expression at later stages. This observation accords with recent evidence that *pitx2* expression in the periocular mesenchyme of zebrafish embryos is altered by the loss of *foxc1a*.^54^ Canonical Wnt signaling also participates in the maintenance of Pitx2 expression by targeting the *Pitx2* promoter,^44^ but Pitx2 expression is upregulated in the corneal stroma of *Dkk2*^−/−^ mice at E16.5,^12^ which suggests that there is a negative feedback loop between Pitx2 and Dkk2. Thus, since both Pitx2 and *Dkk2* levels are downregulated in the corneas of NC-*Foxc1*^−/−^;NC-*Foxc2*^−/−^ mice at E14.5 (Fig. 7), the relationship between Foxc1/c2 and Pitx2 expression does not appear to be mediated by Wnt signaling, which suggests that Foxc1/c2 and Pitx2 likely have different roles during the early stages of eye development, even though some of the downstream components that participate in their respective signaling cascades (e.g., Dkk2) may be the same. Unlike Pitx2 and Foxc1/c2, the Pitx2's effector AP-2β is dispensable for early specification of corneal epithelium and establishment of lymphangiogenic privilege in the cornea,^45^ suggesting phenotypic differences among key regulators expressed in NC-derived ocular mesenchyme. Since previous studies show that FOXC1 coactivates PITX2-targeted genes by physically interacting with PITX2,^55^ further studies are needed to define the molecular interactions between Foxc1/c2 and Pitx2 during eye development.

Corneal neovascularization and conjunctivalization are also dependent on the activity of the canonical Wnt antagonist Dkk2,^12,56^ and lack of *Dkk2* in the corneal mesenchyme causes a switch from a corneal fate to a conjunctival fate in the ocular ectoderm.^11^ These observations would be consistent with the results from previous reports that suggest Wnt signaling plays an important role in ocular surface homeostasis by regulating limbal stem cell proliferation^57^ and corneal epithelium identity.^50^ In conclusion, our findings identify Foxc1 and Foxc2 as key regulators of canonical Wnt signaling in the development and maintenance of the ocular surface including the cornea. Our results not only provide new insight into ocular surface biology, but also are of high clinical significance for the pathology of corneal and ocular surface diseases.

## Supplementary Material

Supplement 1

Supplement 2

Supplement 3

Supplement 4

Supplement 5

Supplement 6
