# Supplementary material for: Foxc1 and Foxc2 in the Neural Crest Are Required for Ocular Anterior Segment Development
Source: Invest Ophthalmol Vis Sci. 2017 Mar;58(3):1368–77. doi: 10.1167/iovs.16-21217 (PMC5361455; doi:10.1167/iovs.16-21217)
Supplement: Supplement 1 [file iovs-58-02-52_s01.pdf]

Supplemental Figure 1

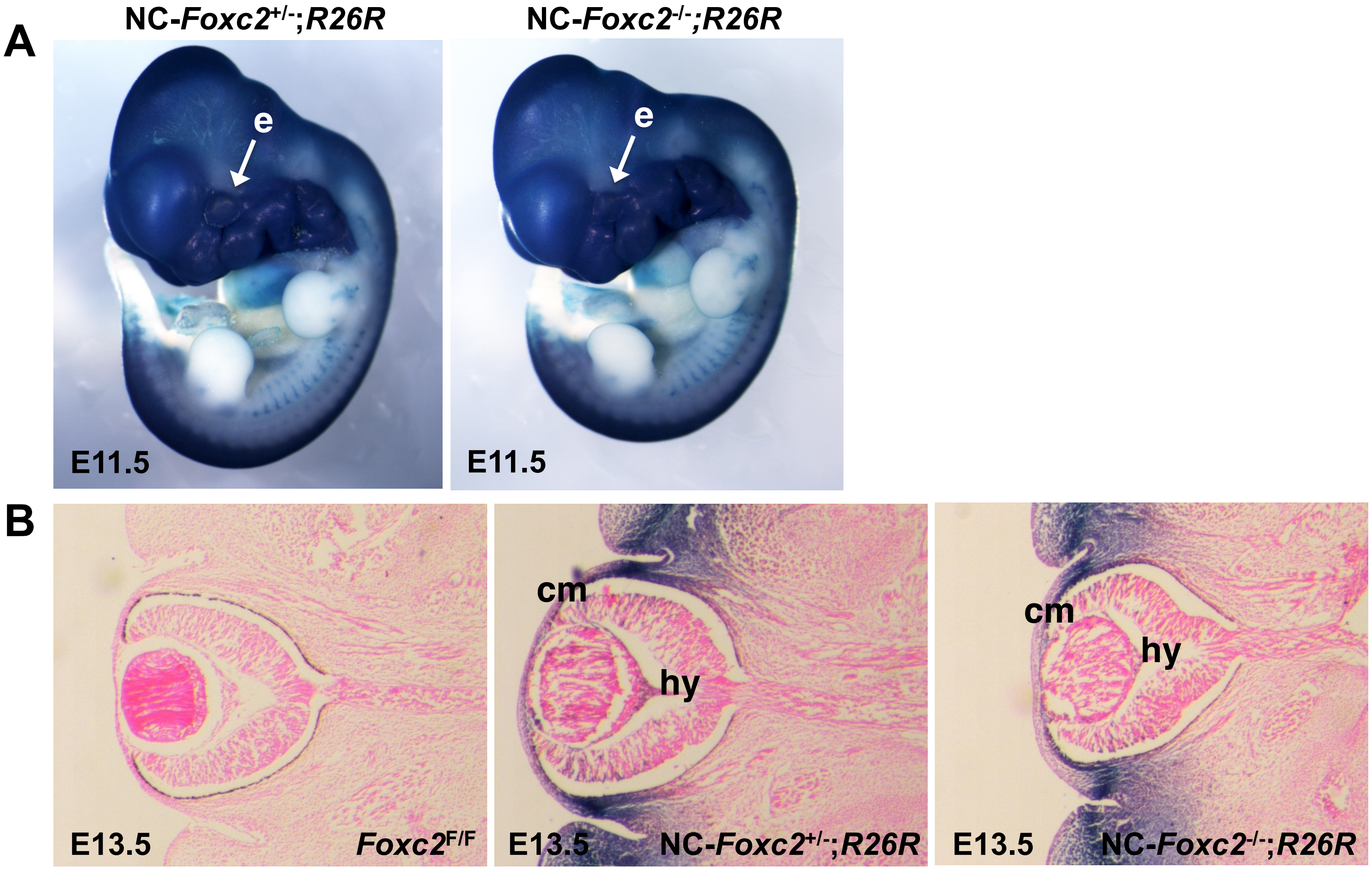

Supplemental Figure 1. NC-specific deletion of *Foxc2* does not alter NC cell migration to the developing eye. Male NC-*Foxc2*$^{+/-}$ mice were bred with female *Foxc2*$^{F/F}$*;R26R* mice carrying a ROSA26-conditional LacZ reporter allele (Rosa26) to generate NC-*Foxc2*$^{+/-}$;*R26R* and NC-*Foxc2*$^{-/-}$;*R26R* embryos. The embryos were subject to X-gal staining to detect NC-derived cells. (A) NC-derived cells were identified in the craniofacial region, the eye (e), the outflow tract of the heart, and the trunk of E11.5 NC-*Foxc1*$^{+/-}$ and NC-*Foxc1*$^{-/-}$ embryos. (B) Within the eyes of E13.5 embryos, NC-derived cells were identified in the corneal mesenchyme (cm) and hyaloid vessels (hy). The results presented in (A) and (B) were consistent with the migration of NC-derived cells in wild-type animals.
