# Supplementary material for: Foxc1 and Foxc2 in the Neural Crest Are Required for Ocular Anterior Segment Development
Source: Invest Ophthalmol Vis Sci. 2017 Mar;58(3):1368–77. doi: 10.1167/iovs.16-21217 (PMC5361455; doi:10.1167/iovs.16-21217)
Supplement: Supplement 2 [file iovs-58-02-52_s02.pdf]

Supplemental Figure 2

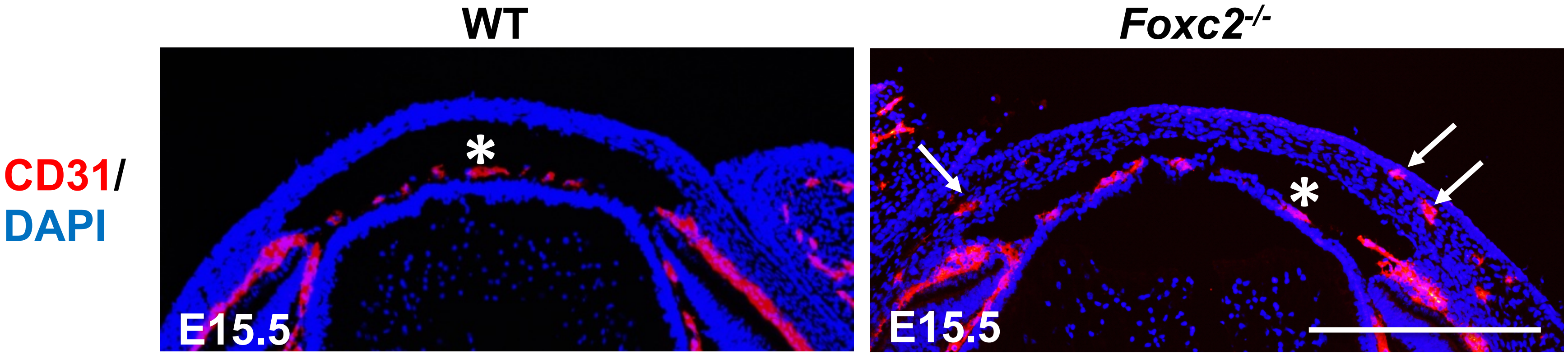

Supplemental Figure 2. Abnormal vessel formation in the cornea of global *Foxc2*$^{-/-}$ mice at E15.5. Immunohistochemistry was performed to detect CD31+ vascular endothelial cells. Global *Foxc2*$^{-/-}$ mutant embryos exhibited abnormal blood vessel formation (arrows) in the peripheral corneal stroma, while they developed the anterior chamber (asterisk) compared to wild-type (WT) embryos. Nuclei were counterstained with DAPI. Scale bar, 100 μm
