# Supplementary material for: Foxc1 and Foxc2 in the Neural Crest Are Required for Ocular Anterior Segment Development
Source: Invest Ophthalmol Vis Sci. 2017 Mar;58(3):1368–77. doi: 10.1167/iovs.16-21217 (PMC5361455; doi:10.1167/iovs.16-21217)
Supplement: Supplement 3 [file iovs-58-02-52_s03.pdf]

Supplemental Figure 3

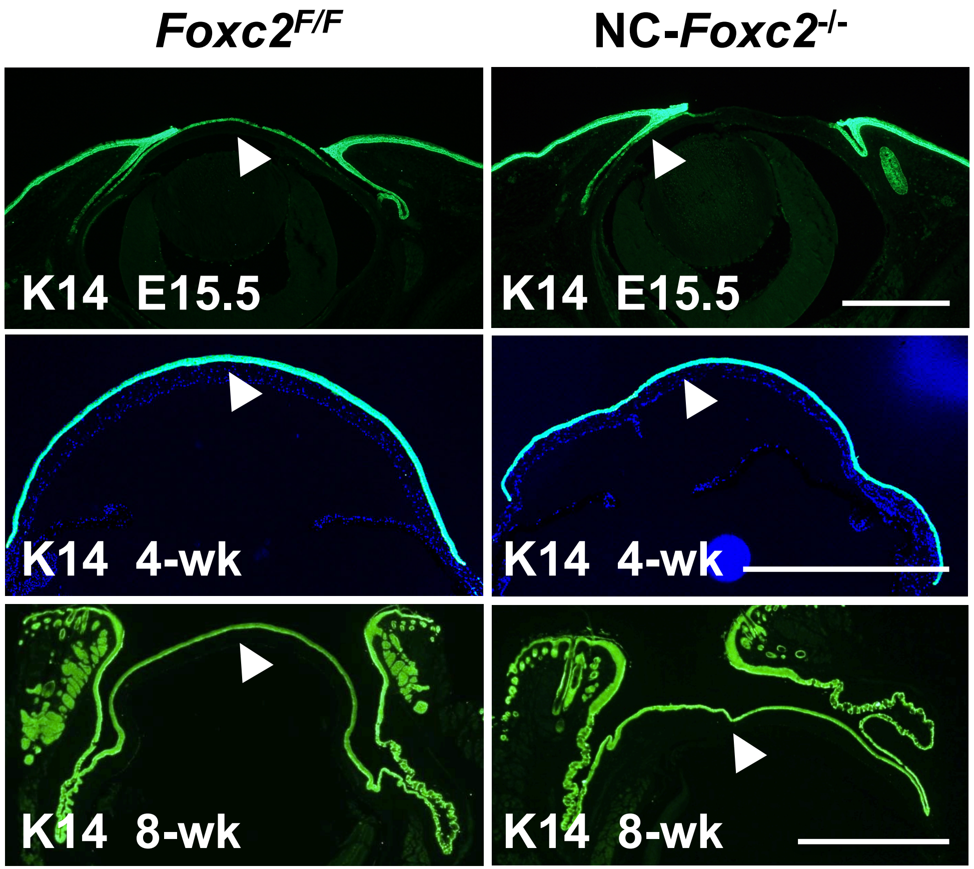

Supplemental Figure 3. Expression of K14 in *Foxc2*$^{F/F}$ and NC-*Foxc2*$^{-/-}$ mice. K14 expression in both *Foxc2*$^{F/F}$ and NC-*Foxc2*$^{-/-}$ mice was not altered in the ocular epithelium including the corneal epithelium (arrowheads). Scale bars, 200 μm.
