# Supplementary material for: Foxc1 and Foxc2 in the Neural Crest Are Required for Ocular Anterior Segment Development
Source: Invest Ophthalmol Vis Sci. 2017 Mar;58(3):1368–77. doi: 10.1167/iovs.16-21217 (PMC5361455; doi:10.1167/iovs.16-21217)
Supplement: Supplement 4 [file iovs-58-02-52_s04.pdf]

Supplemental Figure 4

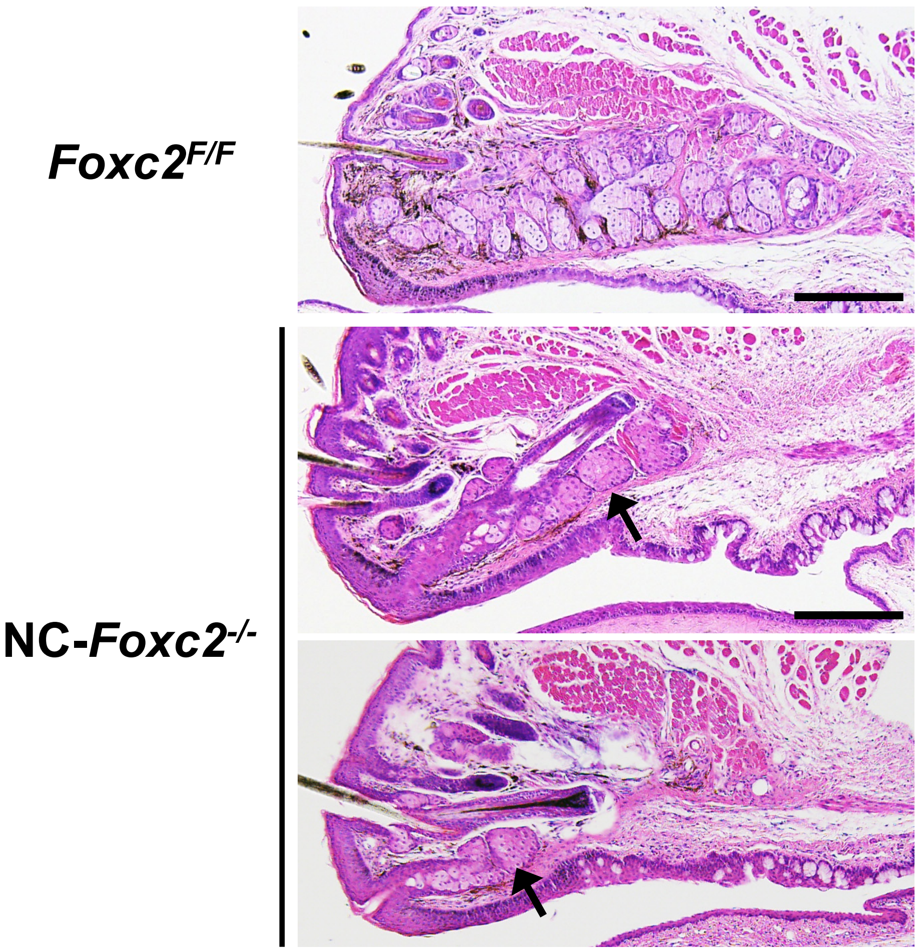

Supplemental Figure 4. Adult NC-*Foxc2*$^{-/-}$ mice display hypoplasia of the meibomian glands.

Arrows indicate hypoplastic meibomian glands in NC-*Foxc2*$^{-/-}$ mice. Scale bars, 100 μm.
