# Supplementary material for: Foxc1 and Foxc2 in the Neural Crest Are Required for Ocular Anterior Segment Development
Source: Invest Ophthalmol Vis Sci. 2017 Mar;58(3):1368–77. doi: 10.1167/iovs.16-21217 (PMC5361455; doi:10.1167/iovs.16-21217)
Supplement: Supplement 5 [file iovs-58-02-52_s05.pdf]

Supplemental Figure 5

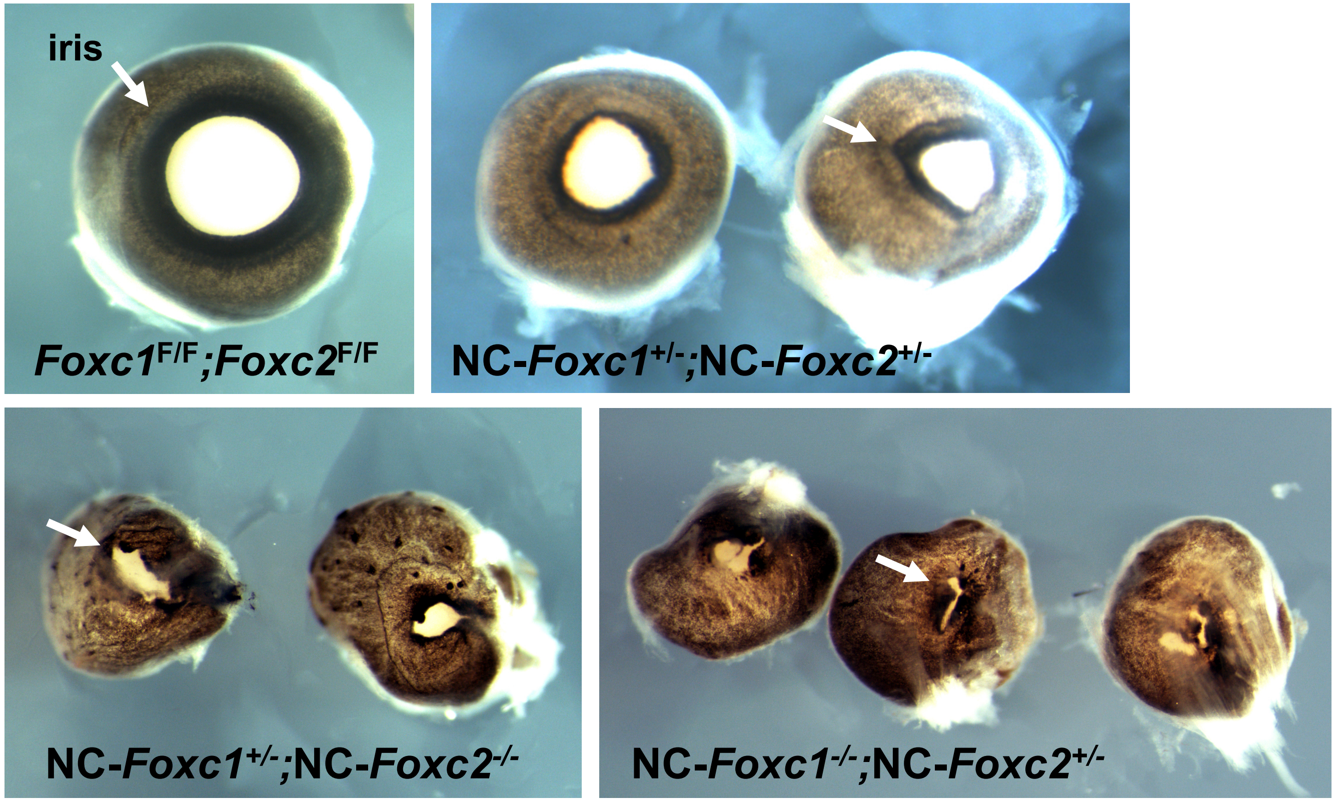

Supplemental Figure 5. The eyeballs of *Foxc1*$^{F/F}$; *Foxc2*$^{F/F}$, NC-*Foxc1*$^{+/-}$;NC-*Foxc2*$^{+/-}$, NC-*Foxc1*$^{+/-}$;NC-*Foxc2*$^{-/-}$, NC-*Foxc1*$^{-/-}$;NC-*Foxc2*$^{+/-}$ mice at P0. Compound NC-*Foxc1*$^{+/-}$;NC-*Foxc2*$^{+/-}$ mice had irregular iris. Compound NC-*Foxc1*$^{+/-}$;NC-*Foxc2*$^{-/-}$ and compound NC-*Foxc1*$^{-/-}$;NC-*Foxc2*$^{+/-}$ mice had smaller eyes, disrupted iris and irregular narrow notched pupil shape (arrows).
