# Supplementary material for: Foxc1 and Foxc2 in the Neural Crest Are Required for Ocular Anterior Segment Development
Source: Invest Ophthalmol Vis Sci. 2017 Mar;58(3):1368–77. doi: 10.1167/iovs.16-21217 (PMC5361455; doi:10.1167/iovs.16-21217)
Supplement: Supplement 6 [file iovs-58-02-52_s06.pdf]

Supplemental Figure 6

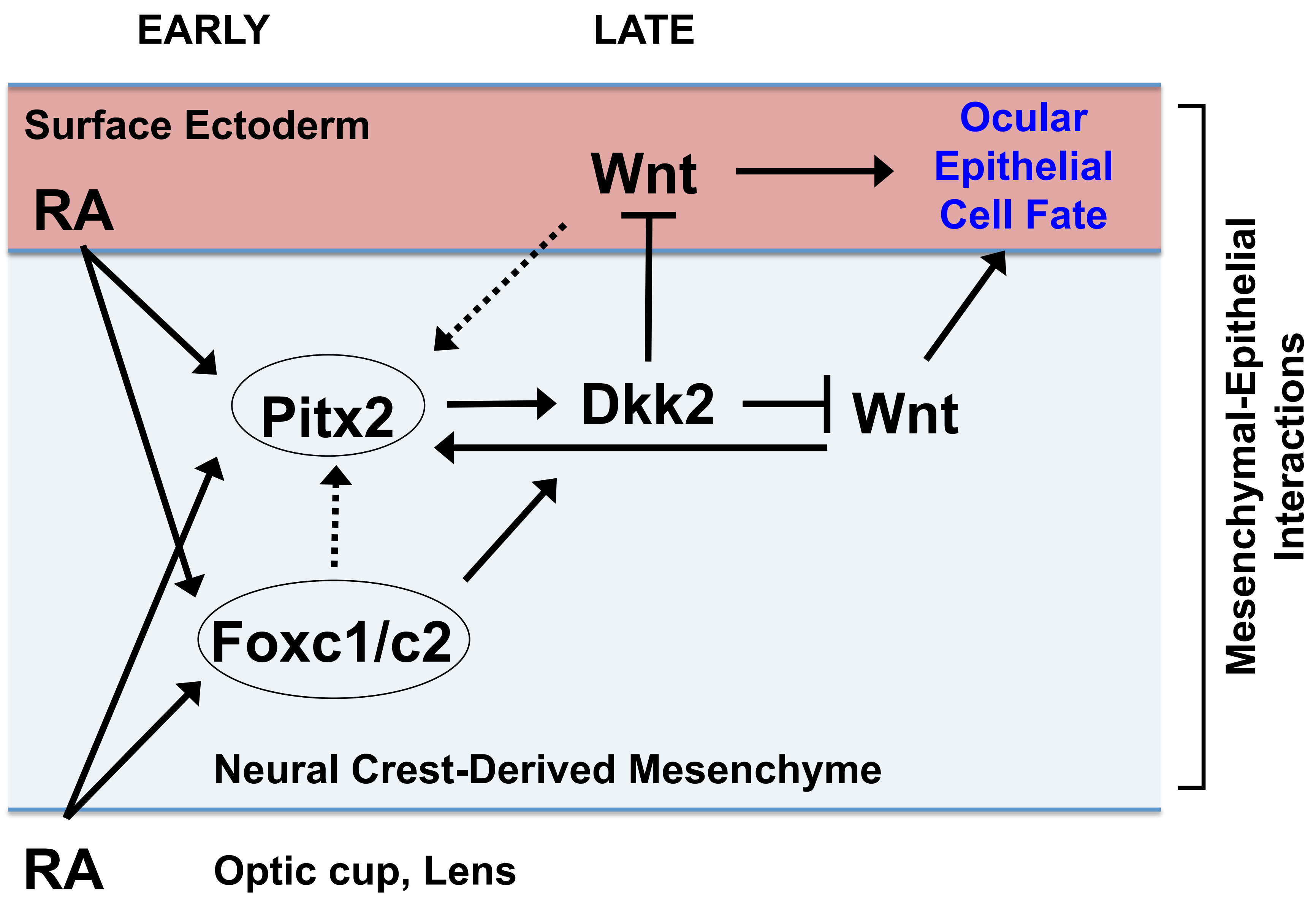

Supplemental Figure 6. Model for the role of Foxc1/c2 during ocular surface development. Retinoic acid (RA) produced from the surface ectoderm, optic cup and lens regulates expression of *Pitx2* and *Foxc1* (and possibly *Foxc2* as well) in the ocular mesenchyme. There are a regulatory loop between *Pitx2* and canonical Wnt signaling. *Foxc1* and *Foxc2* are not required for the induction of *Pitx2* expression, but they are essential for the maintenance of *Pitx2* expression.
